# Selective IKK2 inhibitor IMD0354 disrupts NF-κB signaling to suppress corneal inflammation and angiogenesis

**DOI:** 10.1007/s10456-018-9594-9

**Published:** 2018-01-13

**Authors:** Anton Lennikov, Pierfrancesco Mirabelli, Anthony Mukwaya, Mira Schaupper, Muthukumar Thangavelu, Mieszko Lachota, Zaheer Ali, Lasse Jensen, Neil Lagali

**Affiliations:** 1 Department of Ophthalmology, Institute for Clinical and Experimental Medicine, Faculty of Health Sciences, Linköping University, 58183 Linköping, Sweden; 2 Laboratory of Biomedical Cell Technologies, School of Biomedicine, Far Eastern Federal University, Vladivostok, Russia; 3 Division of Cardiovascular Medicine, Department of Medical and Health Sciences, Linköping University, Linköping, Sweden; 4 Department of Immunology, Medical University of Warsaw, Warsaw, Poland

**Keywords:** Cornea, Neovascularization, NF-κB, IMD0354, IKK2, VEGF

## Abstract

Corneal neovascularization is a sight-threatening condition caused by angiogenesis in the normally avascular cornea. Neovascularization of the cornea is often associated with an inflammatory response, thus targeting VEGF-A alone yields only a limited efficacy. The NF-κB signaling pathway plays important roles in inflammation and angiogenesis. Here, we study consequences of the inhibition of NF-κB activation through selective blockade of the IKK complex IκB kinase β (IKK2) using the compound IMD0354, focusing on the effects of inflammation and pathological angiogenesis in the cornea. In vitro, IMD0354 treatment diminished HUVEC migration and tube formation without an increase in cell death and arrested rat aortic ring sprouting. In HUVEC, the IMD0354 treatment caused a dose-dependent reduction in VEGF-A expression, suppressed TNFα-stimulated expression of chemokines *CCL2* and *CXCL5*, and diminished actin filament fibers and cell filopodia formation. In developing zebrafish embryos, IMD0354 treatment reduced expression of Vegf-a and disrupted retinal angiogenesis. In inflammation-induced angiogenesis in the rat cornea, systemic selective IKK2 inhibition decreased inflammatory cell invasion, suppressed CCL2, CXCL5, *Cxcr2*, and TNF-α expression and exhibited anti-angiogenic effects such as reduced limbal vessel dilation, reduced VEGF-A expression and reduced angiogenic sprouting, without noticeable toxic effect. In summary, targeting NF-κB by selective IKK2 inhibition dampened the inflammatory and angiogenic responses in vivo by modulating the endothelial cell expression profile and motility, thus indicating an important role of NF-κB signaling in the development of pathologic corneal neovascularization.

**Electronic supplementary material:**

The online version of this article (10.1007/s10456-018-9594-9) contains supplementary material, which is available to authorized users.

## Introduction

Corneal neovascularization, the pathological ingrowth of blood vessels into the normally avascular cornea, can lead to tissue scarring, lipid deposition, corneal edema and a profound decline in vision. Moreover, it can worsen the prognosis for corneal transplantation [1, 2]. New blood vessel growth is characteristically driven by a gradient of vascular endothelial growth factor (VEGF)-A, which has led to the hypothesis that anti-VEGF agents could function as an effective treatment strategy for corneal neovascularization [3]. However, the efficacy of anti-VEGF treatments varies among patients and results in only partial vessel regression [4]. In an inflammatory model in the rat, Mirabelli et al. [5] reported that topical anti-VEGF treatment reduces corneal neovascularization by only 14%. Targeting VEGF-A, while addressing the pathologic angiogenic component, does not directly target the associated inflammation. Cross talk between angiogenesis and inflammation is apparent where inflammatory cytokines and chemokines stimulate vessel growth, while new vessels release inflammatory cells to infiltrate the inflammation site. The transcription factor “nuclear factor (NF)-κB” plays an essential role in innate immunity, inflammation, cell survival, cell differentiation and cell proliferation. Studies have reported the role of NF-κB in angiogenesis through its regulation of the inflammatory response and VEGF expression [6, 7]. However, NF-κB-dependent VEGF regulation is not well understood and is reported to be cell or tissue specific [8]. The effect of NF-κB activation appears to depend upon the stimulus, context of activation and cell type [9]. Also, little is known about the possible role of NF-κB in vascular endothelial cells, which could represent an important mediator of cross talk between inflammatory and angiogenic cells and processes.

NF-κB is located in the cytoplasm in its inactive dimeric form and is bound to the regulatory protein inhibitors of κB (IκB) family. Upon stimulation (for instance by inflammatory signals like tumor necrosis factor (TNF)-α), IκB kinase (IKK) complex phosphorylates the inhibitor IκB subunit. This modification marks IκB for degradation and enables nuclear translocation of the free NF-κB [10, 11]. Nuclear NF-κB binds to its target sequence (κB sites) and promotes transcription of a host of target genes, such as TNF-α, chemokine (C–C motif) ligand 2 (CCL2; MCP-1) and C-X-C motif chemokine 5 (CXCL5; ENA78) [12]. These factors can induce monocyte and neutrophil invasion into tissue and may in turn further activate NF-κB signaling through their putative cell surface receptors. In the rat model of corneal angiogenesis, this inflammatory positive feedback loop leads to CD45^+^ cell infiltration into the corneal stroma [13]. Furthermore, it was recently shown that *CCL2* and *CXCL5*, downstream factors of the NF-κB signaling pathway, are among the most up-regulated genes in corneal neovascularization in the presence of inflammation [14].

NF-κB activation is controlled by the IKK complexes that are formed by two kinases [IKK1 (IKKα) and IKK2 (IKKβ)] and a regulatory subunit [IKKγ/NF-κB essential modifier (NEMO)]. Targeting these complexes could serve as a potential means to regulate NF-κB activation. However, knockout of IKK2 and NEMO in mice resulted in embryonic lethality due to massive hepatocyte apoptosis [15]. Furthermore, IKK1 deficient mice exhibit developmental defects and die shortly after birth [16, 17]. Two NF-κB pathways exist; the rapid canonical pathway turned on by pro-inflammatory stimuli associated with IKK2 and NEMO, and the slower IKK1-dependent non-canonical pathway related to lymphoid organogenesis [16]. Inflammation-induced NF-κB activation is associated with the canonical pathway resulting in IκBα phosphorylation through IKK2 [10].

IMD0354 (*N*-(3, 5-bis-trifluoromethyl-phenyl)-5-chloro-2-hydroxy-benzamide) is a non-ATP binding competitive selective IKK2 inhibitor [18–20]. In 2001, the anti-inflammatory effects of IMD0354 were demonstrated by its ability to ameliorate endotoxin-induced uveitis in rats [21]. Furthermore, IMD0354 treatment revealed an inhibitory effect on Vegf expression in a murine model of diabetic retinopathy, while preserving vessel wall integrity [22]. These results were later independently confirmed in ovarian cancer cells [23]. Furthermore, inhibition of NF-κB by IMD0354 has been studied in several preclinical models including cancer [23], reperfusion injury [20], allergy [19] and in lung fibrosis [18].

This study aimed to investigate the potential of selective IKK2 inhibition to suppress inflammation and subsequent corneal neovascularization. Possible effects of IKK2 inhibition were first examined on human umbilical vein endothelial cells (HUVEC) and then on VEGF-A-dependent vasculogenesis in the developing zebrafish embryo. Finally, inhibition of NF-κB was evaluated in an inflammatory corneal neovascularization model in rats, using several measures to assess its anti-inflammatory and anti-angiogenic potential.

## Results

### IKK2 inhibition blocks NF-κB activation in HUVEC

**Fig. 1:**
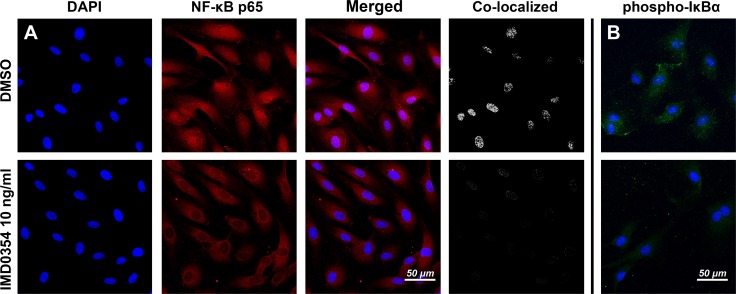
Effect of IMD0354 treatment on NF-κB nuclear translocation and phospho-IκBα expression on 24 h serum-starved HUVEC. Inhibitory effect of IMD0354 (10 ng/ml) on NF-κB (red) nuclear translocation (pink) (**a**), and phospho-IκBα (green) expression (**b**). In both (**a**) and (**b**), IMD0354 is relative to controls (DMSO 1 µl/ml). Cell nuclei visualized with DAPI staining (blue)

The effect of IKK2 inhibition in HUVEC was tested by adding 10 ng/ml of IMD0354 to serum-starved HUVEC cultured for 24 h. Following immunohistochemical analysis of NF-κB p65 (RelA) translocation into the nucleus (Fig. 1a) and analysis of the levels of phosphorylated IκBα (Fig. 1b), it was found that IMD0354 reduced phosphorylation of IκBα, and consistent with this, blocked nuclear translocation of NF-κB p65, thereby suppressing activation of the NF-κB pathway.

### IKK2 inhibition reduces HUVEC migration and tube formation in vitro

As a critical step in new blood vessel formation, endothelial cells migrate to form tubes [24]. Therefore, we investigated whether selective IKK2 inhibition affects migration and tube formation in HUVEC in vitro.

**Fig. 2:**
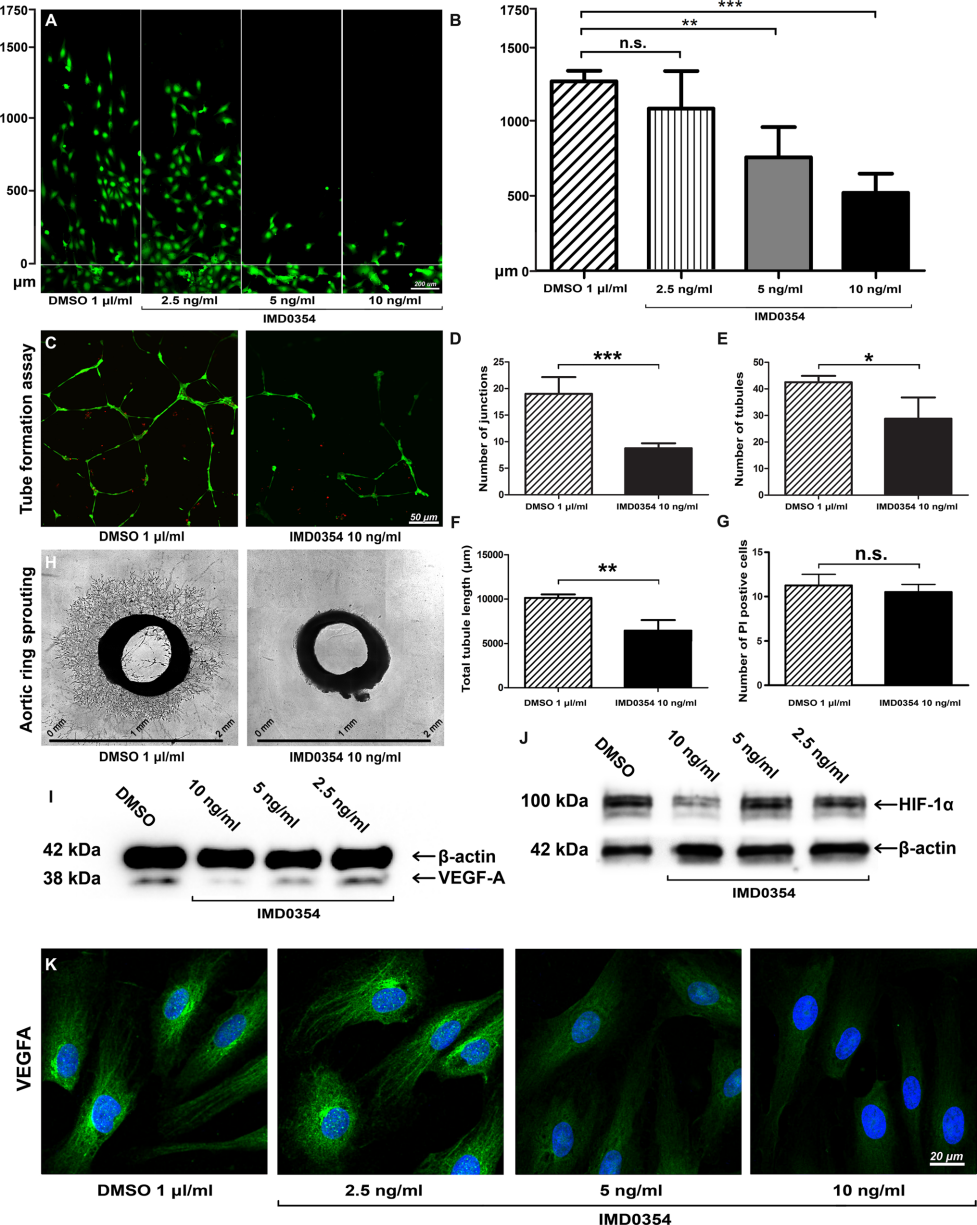
Effect of IKK2 inhibition on HUVEC, microvessel outgrowth from aortic rings, and VEGF-A expression. **a** Dose-dependent (10, 5 and 2.5 ng/ml) inhibitory effect of IMD0354 on HUVEC migration relative to control (DMSO 1 µl/ml). Living HUVEC were visualized with Calcein-AM (green). **b** Quantification of HUVEC migration distance (*n* = 8). One-way ANOVA test with Tukey multiple comparison was used to determine statistical significance. **c** HUVEC has grown on Geltrex to evaluate tube formation in the presence of IMD0354 compared to drug-free vehicle (DMSO). Vital HUVEC are stained with calcein-AM (green), and dead HUVEC are displayed in red (propidium iodide). Quantitative analysis of a number of junctions (**d**), and tubules (**e**) formed by HUVEC, and total tubule length (**f**) (*n* = 8). **g** Quantitative analysis of cell death induced by IMD0354 treatment (*n* = 8). **h** Effect of IMD0354 on cell proliferation in the aortic ring assay. Student *t* test was used to determine statistical significance. **i** Western blot analysis of VEGF-A expression in HUVEC treated with IMD0354 (10, 5, 2.5 ng/ml), with β-actin as a loading control. **j** Western blot analysis of HIF-1α expression in HUVEC treated with IMD0354 (10, 5, 2.5 ng/ml), with β-actin as a loading control. **k** Immunofluorescent detection of VEGF-A (green) in HUVEC treated with IMD0354 (10, 5, 2.5 ng/ml). Cell nuclei visualized with DAPI staining (blue). n.s. *p* > 0.05; *p < 0.05; **p < 0.01; ***p < 0.001

Using the razor scratch wound assay [25], IMD0354-treated HUVEC showed a dose-dependent reduction in migration rate (Fig. 2a). Significant inhibition of migration occurred at IMD0354 concentrations of 10 ng/ml (*p* < 0.001) and 5 ng/ml (*p* < 0.01) but not at 2.5 ng/ml (*p* > 0.05) (Fig. 2b). Next, tubule formation was assessed in two different models; in the first model, the in vitro tube formation assay, HUVEC grown on Geltrex were treated with either 10 ng/ml IMD0354 or DMSO as control (Fig. 2c). Partially disintegrated tube structures, significant reduction in junction formation (*p* < 0.001; Fig. 1d), reduction in tubule formation (*p* < 0.05; Fig. 2e) and decreased total tubule length (*p* < 0.01; Fig. 2f) were observed with IMD0354 treatment, relative to controls. Quantitative analysis of propidium iodide (PI) staining indicated no increased cell death (*p* > 0.05) after IMD0354 (10 ng/ml) treatment (Fig. 2g), suggesting no increased cytotoxicity (Fig. 2g). The second model involved evaluating vessel sprouting ex vivo using the rat aortic ring assay. As shown in Fig. 2h, IMD0354 treatment at 10 ng/ml inhibited an outgrowth of vascular structures from the initial aortic ring. Conversely, aortic rings cultured with drug-free vehicle (DMSO) exhibited a radial outgrowth of numerous vascular tubule structures.

### IKK2 inhibition down-regulates VEGF-A and HIF-1α expression in HUVEC

In addition to NF-κB signaling blockade, we investigated a possible direct anti-angiogenic effect of IKK2 inhibition by studying VEGF-A expression. Western blot analysis on HUVEC treated with IMD0354 at 10, 5 and 2.5 ng/ml indicated a dose-dependent decrease in VEGF-A expression, most prominent at 10 ng/ml (Fig. 2i). HIF-1α expression was decreased at 10 ng/ml, but not at lower concentrations (Fig. 2j). Immunofluorescent detection of VEGF-A expression in HUVEC confirmed Western blot findings (Fig. 2k), suggesting that IKK2 inhibition reduces HIF-1α and VEGF-A expression in HUVEC.

### IKK2 inhibition disrupts HUVEC migration modulating the cytoskeleton and cell filopodia formation

**Fig. 3:**
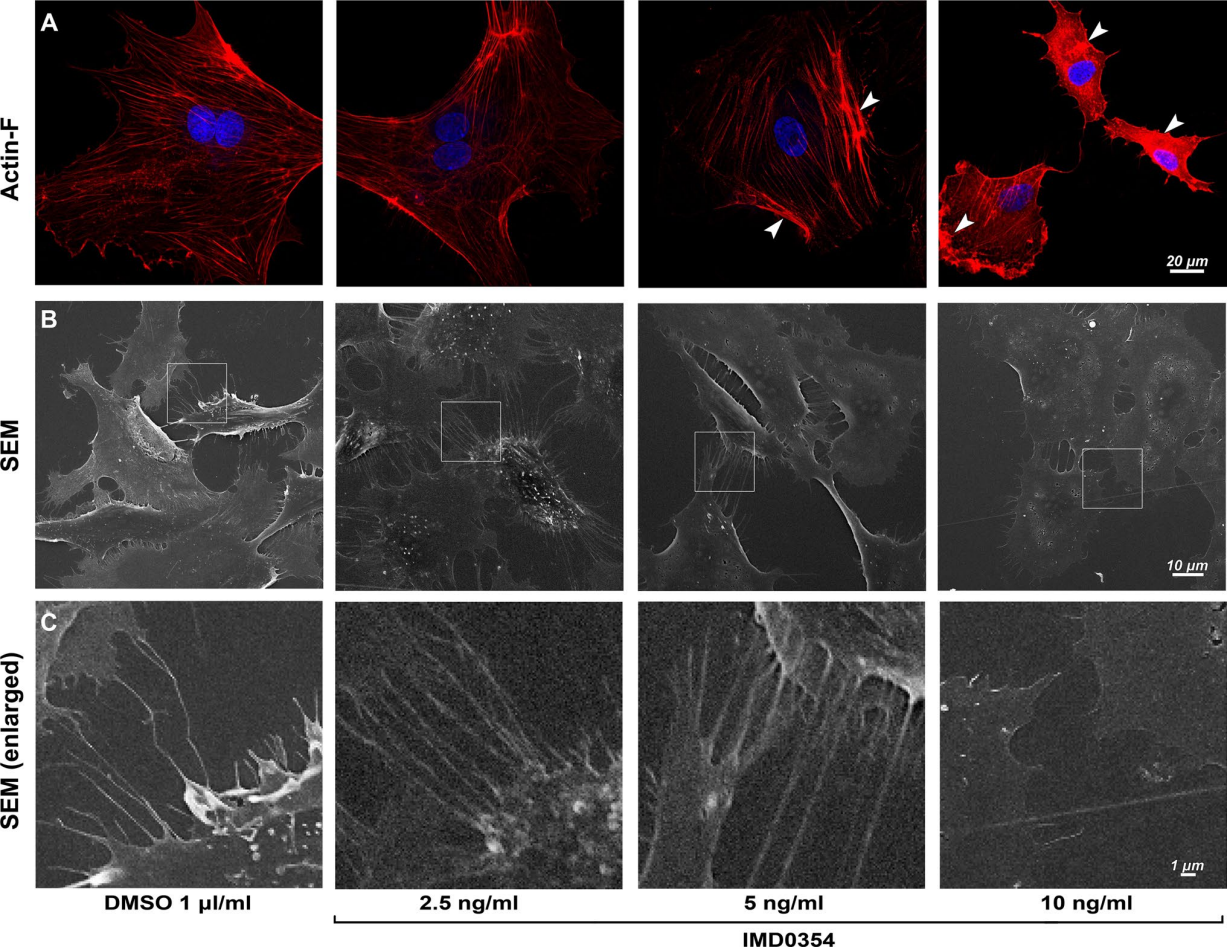
IKK2 inhibition disrupts HUVEC cytoskeleton, filopodia structure development and cell-to-cell interaction. **a** Impact of IMD0354 on HUVEC cytoskeleton arrangement examined by phalloidin staining (red), Actin-F aggregates in the cytoplasm of HUVEC is indicated by arrows. Cell nuclei visualized with DAPI staining (blue). **b** Scanning electron microscopy (SEM) images of HUVEC surface and cellular filopodia when treated with IMD0354 (10, 5, 2.5 ng/ml). **c** Enlarged SEM images indicate changes in cell-to-cell filopodia interactions with IMD0354 treatment

We examined a possible mechanism by which HUVEC migration is disrupted by IKK2 inhibition. Migration of cells is driven by coordinated changes in microfilament network formation, where Actin-F is a key structural protein for this process. We investigated the influence of IKK2 inhibition on the HUVEC cytoskeleton, by visualizing the Actin-F cytoskeleton stained with phalloidin red (Fig. 3a). A disruptive effect on Actin-F cytoskeleton formation was evident with IMD0354 at 10 ng/ml (Fig. 3, arrows); here, Actin-F failed to produce well-defined cytoskeletal structures and remained aggregated in the cellular cytoplasm. In parallel with the cytoskeletal reorganization, the appearance of cell filopodia is a feature of migrating cells. By scanning electron microscopy (SEM), a notable reduction in the appearance cell filopodia was observed (Fig. 3b). Enlarged areas of SEM images (Fig. 3c) demonstrated a reduced cell-to-cell filopodia interaction in treated cells.

### IKK2 inhibition suppresses expression of inflammatory chemokines CCL2 and CXCL5 in TNF-α-stimulated HUVEC

**Fig. 4:**
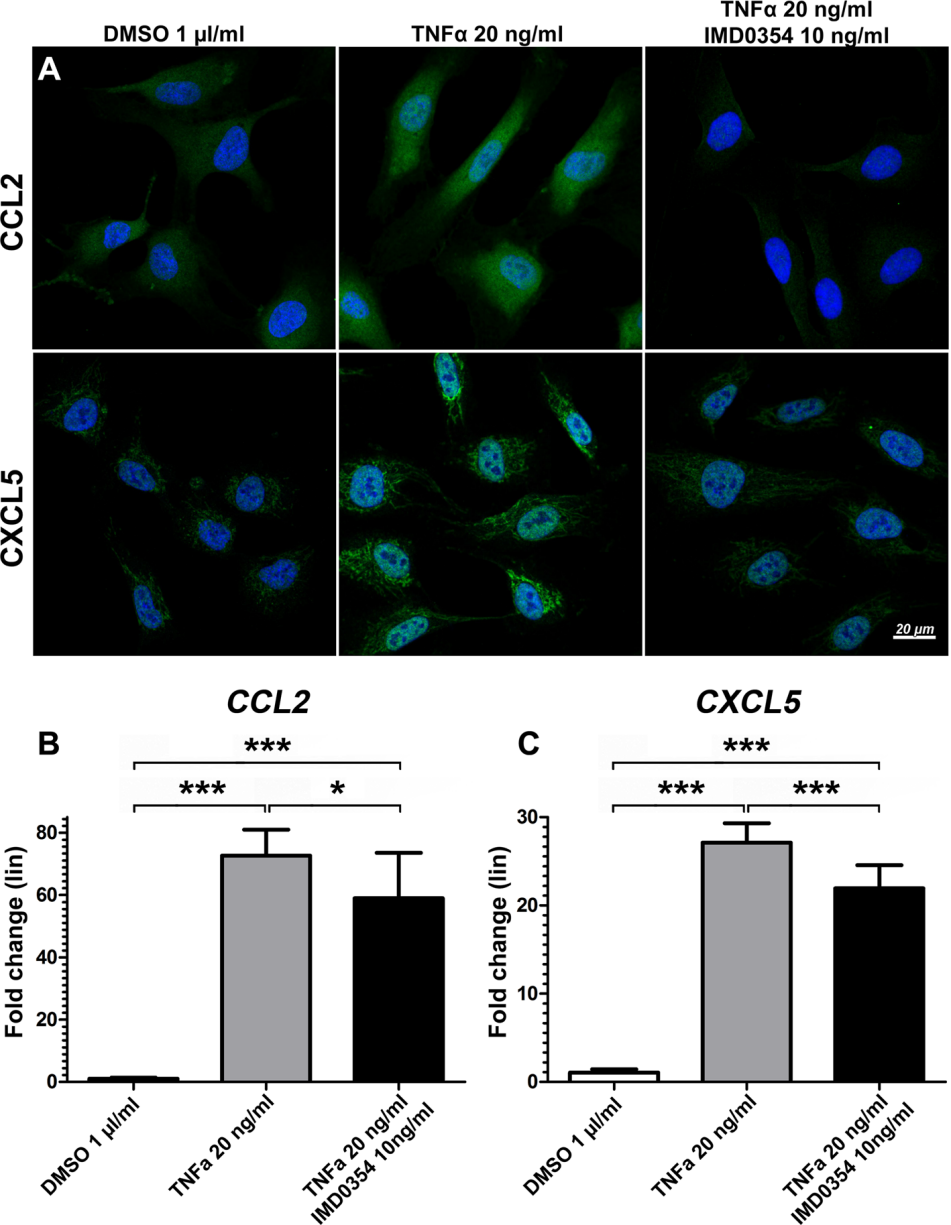
Effects of IKK2 inhibition on CCL2 and CXCL5 expression in HUVEC under TNFα stimulation. **a** Immunofluorescence images of CCL2 (green) and CXCL5 (green) treated with IMD0354 and stimulated by recombinant human TNF-α (rhTNFα). DMSO and rhTNF-α treatment were used as a negative and positive control, respectively. Cell nuclei visualized with DAPI staining (blue). Quantitative qRT-PCR analysis of **b** *CCL2* and **c** *CXCL5* expression in IMD0354 treated and rhTNF-α stimulated HUVEC (*n* = 4). One-way ANOVA test with Tukey multiple comparisons was used to determine statistical significance. *p < 0.05; ***p < 0.001

HUVEC can express both CCL2 [26] and CXCL5 [27] to enhance the inflammatory response. To clarify the role of endothelial cells in the production of these potent pro-inflammatory chemokines, HUVEC were stimulated with recombinant human TNF-α (rhTNFα). IKK2 inhibition affected CCL2 and CXCL5 expression which was examined by antibody staining (Fig. 4a) and qRT-PCR (Fig. 4b, c). A basal level of expression of CCL2 and CXCL5 in quiescent HUVEC was observed in DMSO-treated negative controls, whereas 20 ng/ml rhTNF-α markedly increased the expression of CCL2 and CXCL5 in HUVEC. This up-regulation was reduced by IMD0354 treatment at 10 ng/ml. qRT-PCR analysis indicated enhanced CCL2 and CXCL5 gene expression levels under rhTNFα stimulation (*p* < 0.01) and reduced gene expression of CCL2 (*p* < 0.05) and CXCL5 (*p* < 0.01) upon IMD0354 treatment. IKK2 inhibition, however, did not completely revert chemokine expression to the basal level as the expression of CCL2 and CXCL5 was still elevated relative to DMSO-treated negative controls (*p* < 0.01).

### IKK2 inhibition impairs vasculogenesis in zebrafish embryo

Zebrafish models are convenient for drug testing and angiogenesis studies due to their optical transparency and rapid embryogenesis. Additionally, their angiogenic process is similar to other vertebrates [28, 29]. To investigate whether effects by IKK2 inhibition observed in HUVEC could be recapitulated in vivo, zebrafish embryos were treated with various concentrations of IMD0354 at 0–72 h post-fertilization (hpf).

**Fig. 5:**
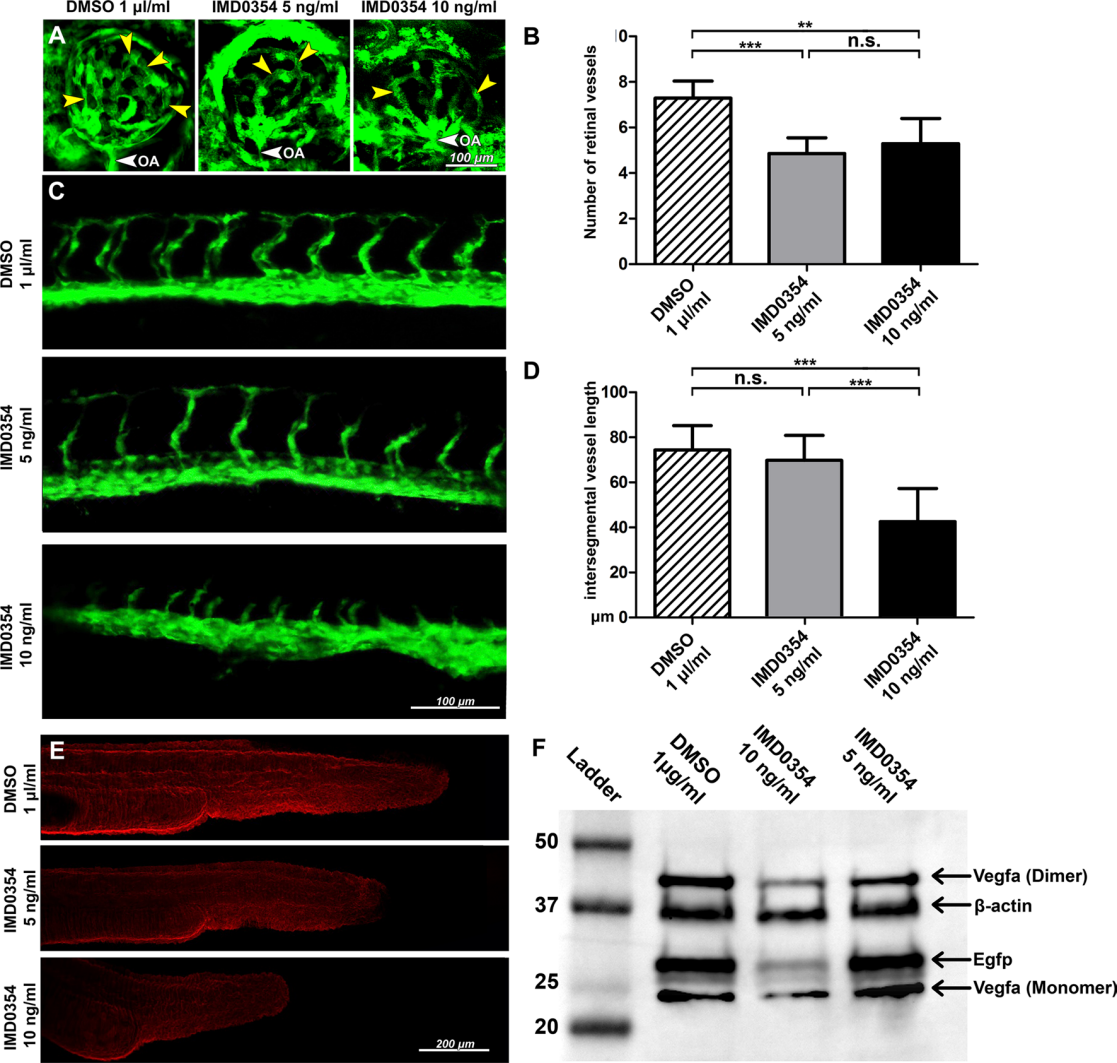
Development of retinal, intersegmental vasculature and expression of Vegf-a and EGFP in 24 h post-fertilization (hpf) Tg(fli1:EGFP)y^1^ zebrafish embryos treated with IMD0354. **a** Detection of EGFP signal from Tg(*fli1*:EGFP)y^1^ transgenic zebrafish embryos retinal vasculature at 72 hpf treated with DMSO or IMD0354 (5, 10 ng/ml). Yellow arrows indicate the retinal vessels. White arrow indicates OA (optic artery). **b** Quantification of the number of retinal vessels at 72 hpf with DMSO or IMD0354 treatment (5 and 10 ng/ml); (*n* = 7) One-way ANOVA test with Tukey multiple comparisons was used to determine statistical significance. **c** Detection of EGFP signal from Tg(*fli1*:EGFP)y^1^ transgenic zebrafish embryos intersegmental vasculature at 28 hpf treated with DMSO or IMD0354 (5 and 10 ng/ml). **d** Quantification of intersegmental vessel length (*n* = 16) One-way ANOVA test with Tukey multiple comparisons was used to determine statistical significance. **e** Immunofluorescent detection of Vegf-a (red) expression in zebrafish embryos at 24 hpf. **f** Western blot analysis of Vegf-a (monomeric and dimeric forms) and Egfp expression in the whole lysate of Tg(fli1:EGFP)y^1^ transgenic zebrafish embryos at 24 hpf, incubated with DMSO and IMD0354 (10 and 5 ng/ml). β-Actin as the loading control. n.s. *p* > 0.05; ***p < 0.001

Retinal vessel development was assessed by confocal fluorescent imaging (Fig. 5a) and quantification (Fig. 5b) at 72 hpf which demonstrated a significant underdevelopment of the retinal vasculature (*p* < 0.001; Fig. 5b) upon IMD0354 (5 and 10 ng/ml) treatment compared to DMSO controls. Effects of IMD0354 treatment on intersegmental vessel development at 28 hpf (Fig. 5c) revealed a significant reduction in intersegmental vessel length at 10 ng/ml (*p* < 0.001; Fig. 5d), whereas 5 ng/ml treatment did not significantly affect intersegmental vasculature development (*p* > 0.05). Immunofluorescence (Fig. 5e) indicated slightly decreased Vegf-a expression in zebrafish at 5 ng/ml concentration and a more prominent inhibition at 10 ng/ml. Western blot (Fig. 5f) revealed a slight (5 ng/ml) and clear (10 ng/ml) reduction in Vegf-a, and overall vasculature through signal reduction in EGFP tagged endothelial cells. Collectively, these findings indicate that IKK2 inhibition can reduce Vegf-a expression and delay zebrafish retinal and intersegmental vasculature development.

### IKK2 inhibition suppresses inflammatory cell infiltration, limbal vasodilation and decreases neovessel density in vivo in the rat cornea

Next, we evaluated the effects of selective NF-κB inhibition on inflammation-induced angiogenesis in the rat cornea. Inflammation leading to corneal neovascularization was induced in Wistar rats by placing two sutures in the cornea of the right eye (time point, 0 h). Rats were treated systemically with IMD0354 (30 mg/kg) or with a vehicle (carboxymethyl cellulose, CMC) immediately after suture placement, and again after 48 h.

**Fig. 6:**
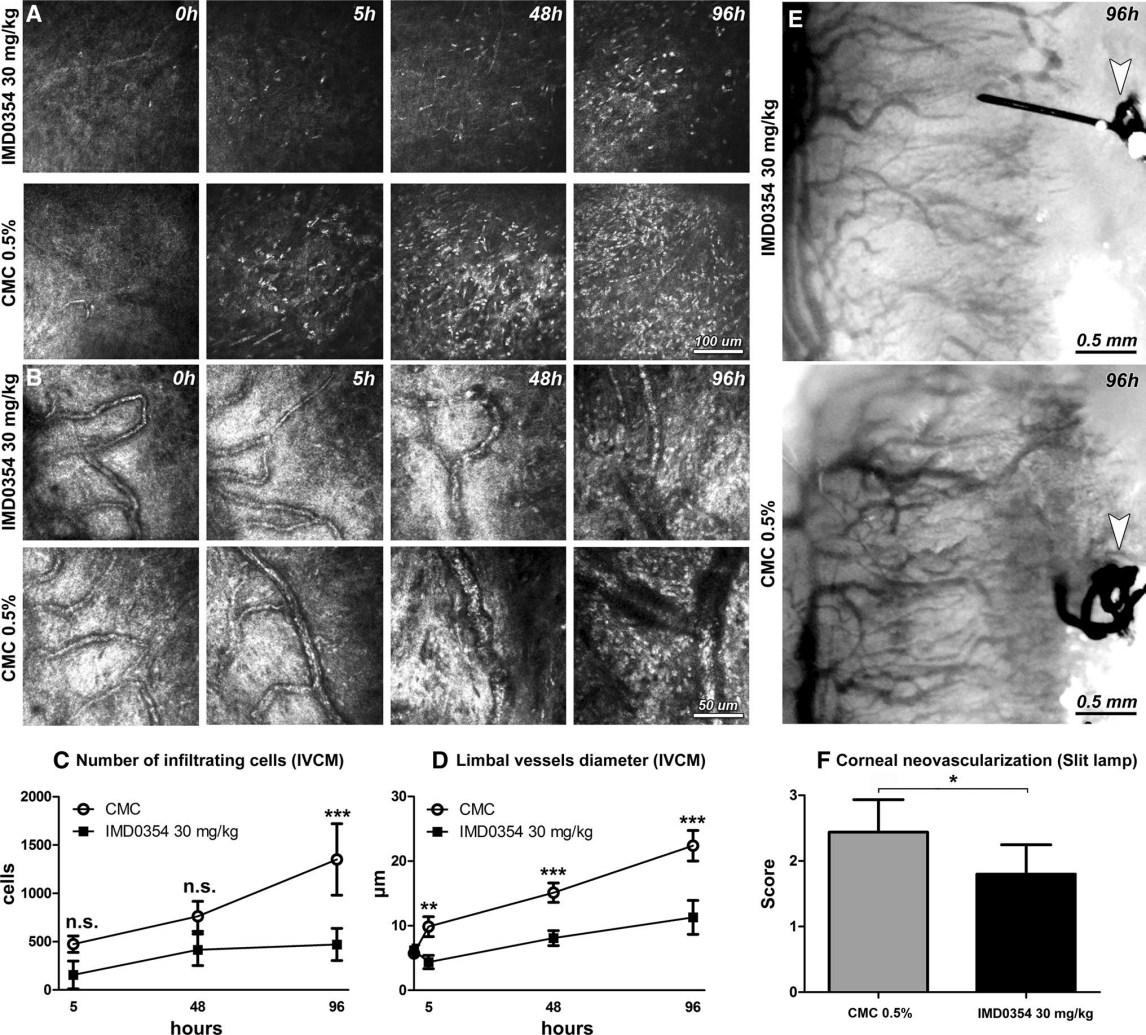
In vivo confocal microscopy (IVCM) of rat cornea after induction of corneal neovascularization by suture placement. IVCM images depicting corneal stromal inflammatory cell infiltration (**a**) and limbal vessels (**b**) in sutured rat corneas at 0, 5, 48 and 96 h. Quantification of infiltrating cells (**c**) and limbal vessel diameter (**d**) in a 400 × 400 μm area (*n* = 8 animals/time point for both). Two-way ANOVA with Bonferroni comparison was used to determine statistical significance. Slit-lamp images of neovascularization of sutured rat corneas at 96 h, treated either with IMD0354 30 mg/kg or CMC (control) (**e**). The arrows point to the suture placed into the cornea. Semi-quantitative vascular density and vascular progression score **(f)**. (*n* = 5 IMD0354 treated; *n* = 8 CMC control group). Student’s *t* test was used to determine statistical significance. n.s. *p* > 0.05; *p < 0.05; **p < 0.01; ***p < 0.001

Inflammatory cell invasion was investigated using in vivo confocal microscopy (IVCM) (Fig. 6a). A substantial number of early migrating inflammatory cells into the corneal stroma were detected in sutured groups. IMD0354 (30 mg/kg) significantly reduced the number of infiltrating inflammatory cells at all examined time points [5 h (*p* < 0.001), 48 h (*p* < 0.01), and 96 h (*p* < 0.001)] (Fig. 6c). Next, we evaluated the diameter of limbal vessels to assess vasodilation as a measure of angiogenic response in the cornea (Fig. 6b). A significant reduction in limbal vasodilation with IMD0354 treatment [5 h (*p* < 0.01), 48 h (*p* < 0.01), and 96 h (*p* < 0.01)] (Fig. 6d) was observed. Phenotype analysis was performed using slit-lamp microscopy in vivo at 96 h (Fig. 6e), and the acquired images were evaluated using a defined neovascularization score. The analysis revealed an overall low corneal neovascularization response (*p* < 0.05) in the IMD0354 treated group (Fig. 6f). The neovascularization score is based on a group of parameters including vessel length (distance from the limbal vessel arcade towords central cornea), vessel caliber (corneal vessel diameter and density) and invasion area (fraction of the corneal area in which vessels are present) (Supplementary Fig. 1) [30].

### IMD0354 reduces nuclear translocation of NF-κB and IκBα phosphorylation in the rat cornea

**Fig. 7:**
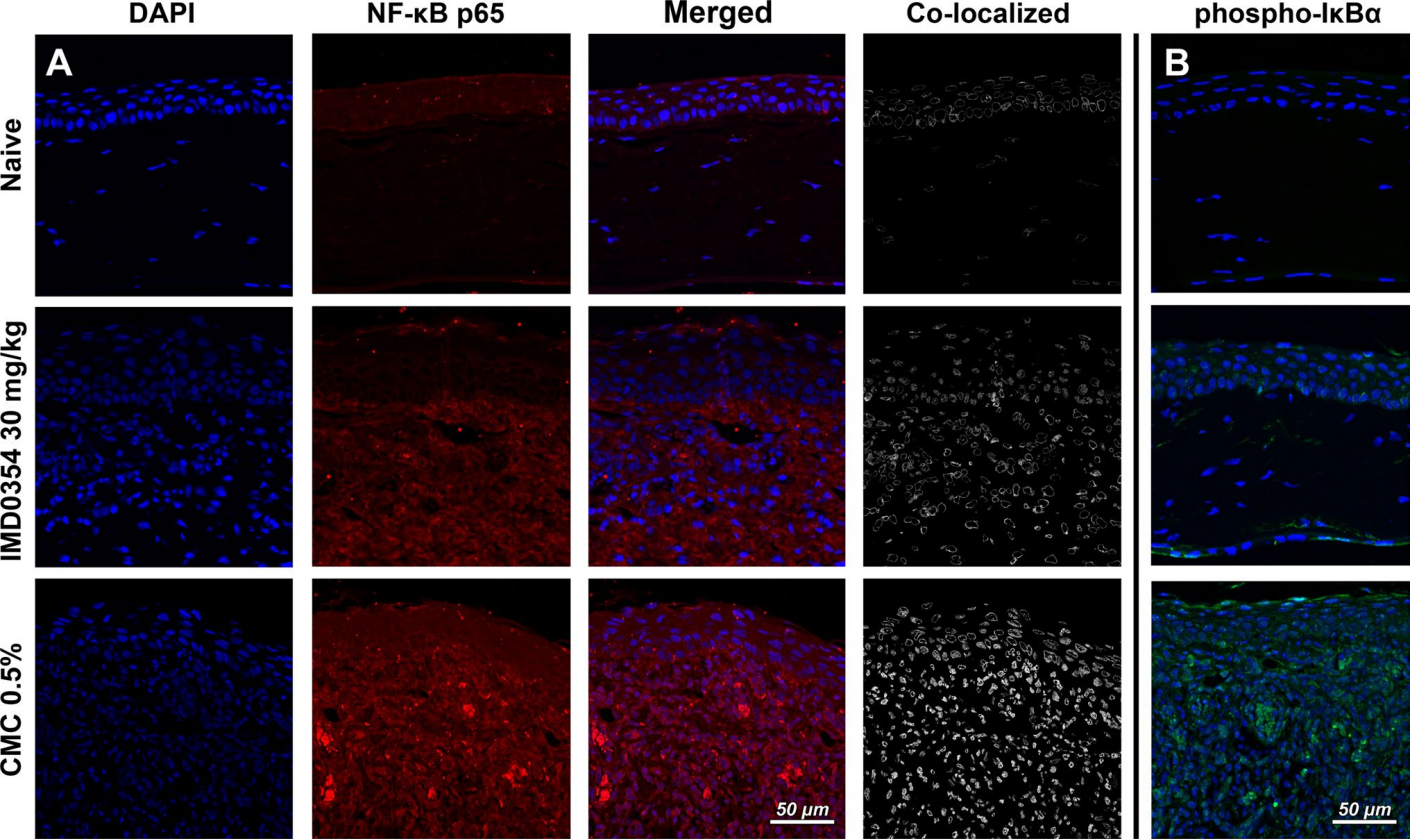
Effect of IMD0354 treatment on nuclear translocation of NF-κB and IκBα phosphorylation in suture-stimulated rat cornea. **a** Inhibitory effect of IMD0354 (30 mg/ml) on NF-κB nuclear translocation and phospho-IκBα expression **b** in suture-stimulated rat cornea at 96 h, relative to control (CMC 0.5%)

NF-κB inhibition by IMD0354 was verified in the rat corneal suture model; immunohistochemistry showed reduced nuclear translocation of NF-κB protein p65 (RelA) (Fig. 7a, b). The decreased signal from NF-κB p65 in the nucleus indicates NF-κB inhibition. The observation of attenuated levels of phosphorylated IκBα in the IMD0354 group provided further evidence of blockade of NF-κB activation.

### IKK2 inhibition attenuates the expression of angiogenic factors and NF-κB downstream inflammatory mediators in the rat cornea

**Fig. 8:**
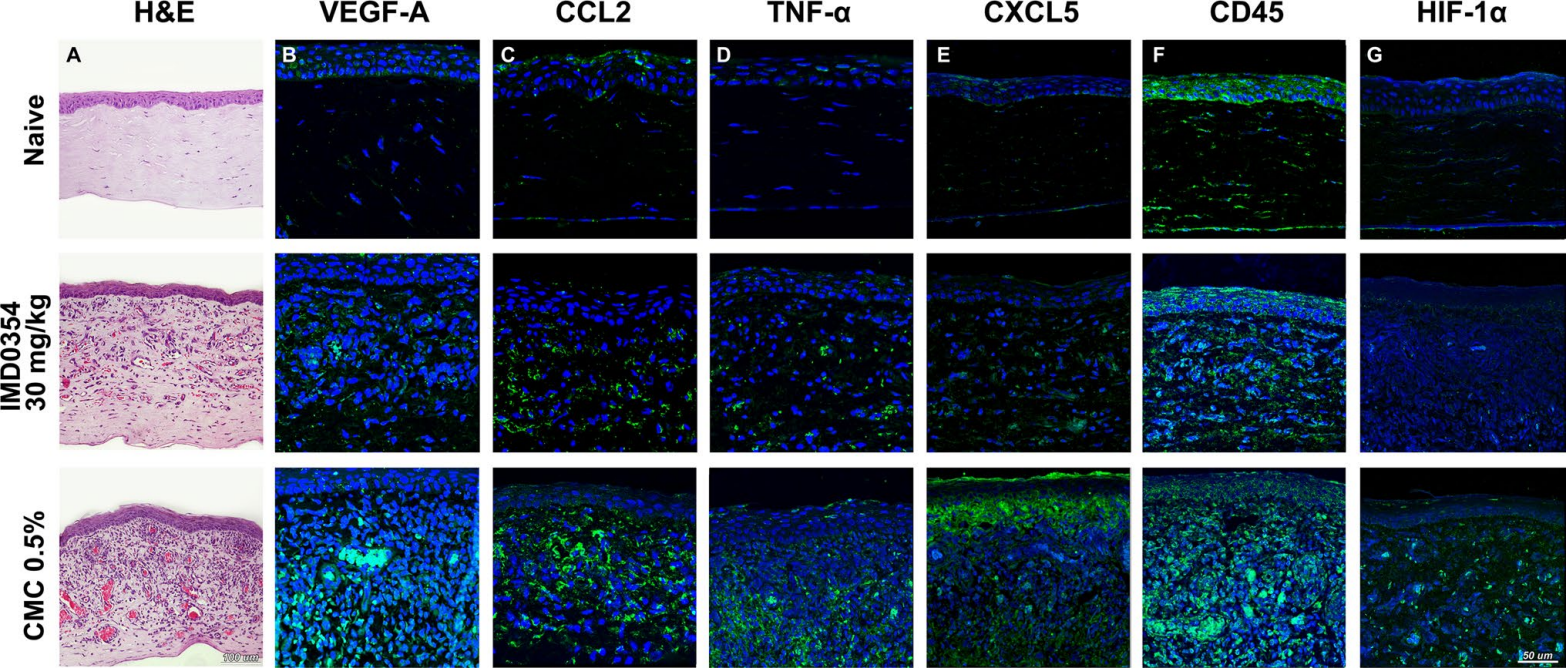
Histology and immunofluorescence in rat corneal sections. **a** Hematoxylin and Eosin (H&E), **b** VEGF-A (green); **c** CCL2 (green); **d** TNF-α (green); **e** CXCL5 (green); **f** CD45 (green) and **g** HIF-1α (green) staining in rat cornea tissue. Nuclear counterstaining by DAPI (blue) in fluorescent images

**Fig. 9:**
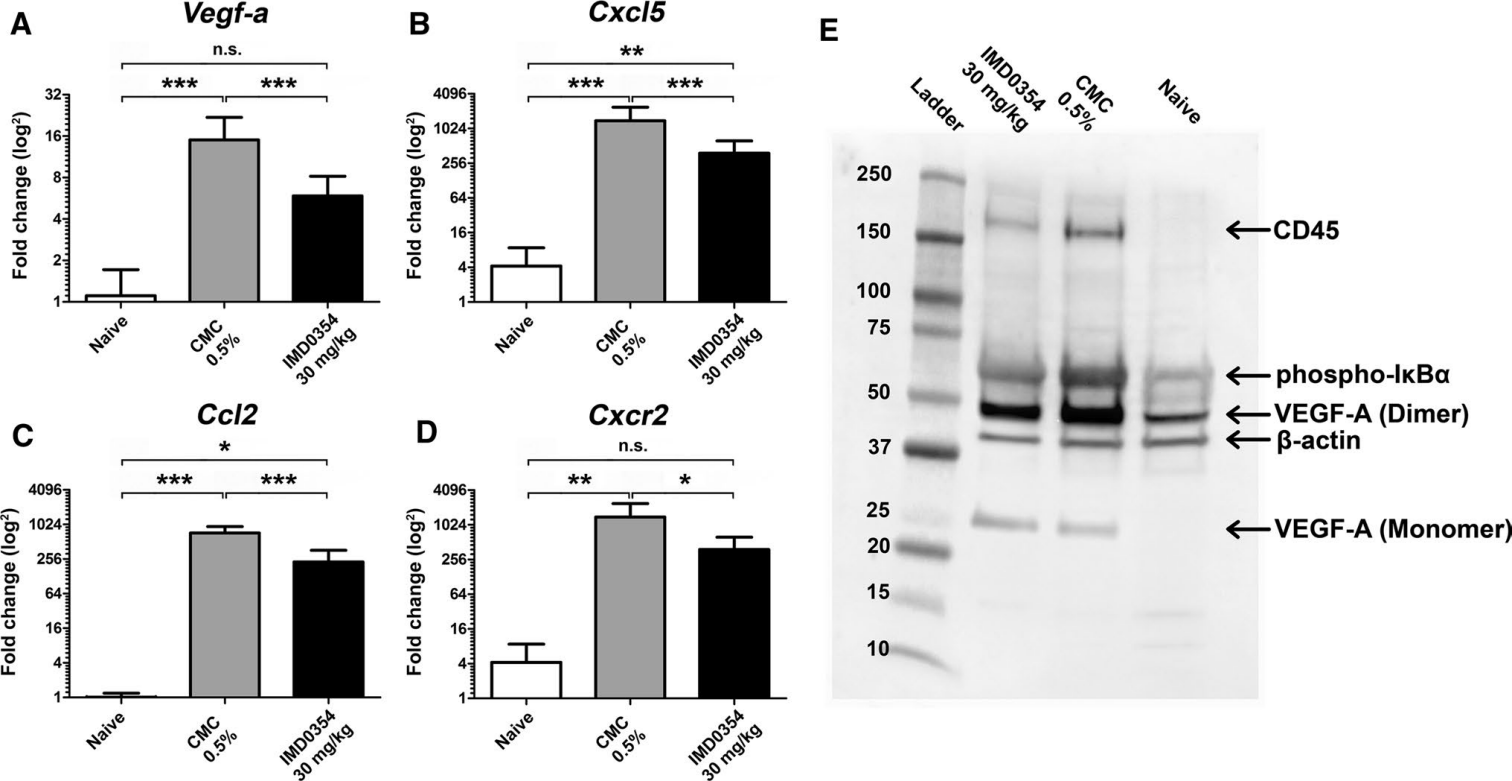
Quantitative qRT-PCR and Western blot analysis of inflammatory and neovascular factors in suture-stimulated rat corneas at 96 h. Quantitative qRT-PCR analysis of **a** *Vegf*-*a*, **b** *Cxcl5*, **c** *Ccl2*, and **d** *Cxcr2* expression in rat cornea. (*n* = 5) One-way ANOVA test with Tukey multiple comparisons were used to determine statistical significance. **e** Western blot analysis of CD45, phospho-IκBα and VEGF-A expression in rat cornea lysate. β-Actin used as a loading control. n.s. *p* > 0.05; *p < 0.05; **p < 0.01; ***p < 0.001

Hematoxylin and eosin (H&E) staining of corneal tissue demonstrated milder cellular infiltration and neovessel formation in the IMD0354 group compared to the control group (Fig. 8a). Tissue stainings for Vegf-A (Fig. 8b), Ccl2 (Fig. 8c), Tnf-α (Fig. 8d), Cxcl5 (Fig. 8e), CdD45 (Fig. 8f) and HIF-1α (Fig. 8g) indicated lower levels of all these proteins in the IMD0354 group relative to the CMC controls. Both groups exhibited higher levels compared to the naive (non-sutured) controls, for all examined factors. Immunofluorescence data were supported by qRT-PCR and Western blot analysis. qRT-PCR indicated a significant down-regulation of the expression of *Vegf*-*a* (*p* < 0.001); (Fig. 9a), *Cxcl5* (*p* < 0.001); (Fig. 9b), *Ccl2* (*p* < 0.001); (Fig. 9c) and *Cxcr2* (*p* < 0.05); (Fig. 9d) in the IMD0354 group compared with CMC controls. The reduction in VEGF-A levels and IκBα phosphorylation was further confirmed by Western blot analysis (Fig. 9e). Furthermore, a reduced protein level of the immune cell marker CD45 was detected in the IMD0354 treated group, relative to the control (CMC).

### Testing of systemic IMD0354 toxicity and teratogenic effects in zebrafish embryos and adult rat liver

No noticeable toxic effect in zebrafish embryos was observed with IMD0354 treatment at concentrations between 1 and 100 ng/ml, at 24 hpf. However, at 200 ng/ml, fish egg development was arrested at 5–6 hpf (Supplementary Fig. 2A). At 72 hpf, 200 ng/ml IMD0354 induced malformations in body shape and arrested nervous system development with no apparent head, eyes or spinal cord. A partially developed cardiovascular system was indicated by a beating heart and a network of misdirected vessels around the yolk sack. At a dose of 100 ng/ml, IMD0354 induced transitory pericardial edema at 72 h that resolved during further development (Supplementary Fig. 2B).

IMD0354 at concentrations of 1–100 ng/ml did not increase embryonic mortality relative to vehicle (*p* > 0.05) at all evaluated time points. At 200 ng/ml, however, 80% of embryos died within the first 24 hpf (*p* < 0.01) with less than 10% surviving to 72 hpf (*p* < 0.01); (Supplementary Fig. 2C). When 200 ng/ml of IMD0354 was first applied to 72 hpf old zebrafish embryos, no lethality or abnormalities were observed across 40 embryos studied to the 5 days post-fertilization (dpf) experimental endpoint.

When IMD0354 was applied systemically in adult Wistar rats, no abnormal hepatic architecture or increased caspase-3-dependent apoptosis was detected (Supplementary Fig. 2D, E). During the experiments, treated rats did not exhibit any abnormal movement or behavior that would be suggestive of neurological complications.

## Discussion

Currently, anti-VEGF therapies are the main treatment strategy for ocular neovascular diseases, where VEGF blockade with monoclonal antibodies (ranibizumab and bevacizumab), as well as newer fusion proteins (aflibercept), has been the standard of care for neovascular AMD during the last decade [31, 32]. Recent clinical studies have shown anti-VEGF potential in reversing retinal vessel proliferation in diabetic retinopathy [33]. By contrast, anti-VEGF agents are less effective in the treatment of corneal angiogenesis, both in clinical settings as well as in experimental models [34]. Inefficacy of VEGF blockade as a treatment strategy may be attributed to VEGF-independent pathways, as we recently reported [13]. Inflammation can trigger the development of corneal neovascularization [35, 36]. Stromal invasion of inflammatory myeloid-lineage cells such as neutrophils and monocytes occurs in the cornea before vessel ingrowth, and these cells promote the release of pro-angiogenic factors [5, 35, 37]. Crucial to inflammation is the transcription factor NF-κB, and its positive feedback which regulates the expression of many cytokines involved in the inflammatory process [38]. Here, administration of the selective IKK2 inhibitor IMD0354 in the rat cornea emphasized inflammatory and angiogenic roles of the NF-κB pathway. IMD0354 treatment reduced pre-angiogenic tissue activity such as limbal vessel dilation and inflammatory cell infiltration into the stroma. Limbal vessels dilate in response to increased VEGF as part of the inflammatory response preceding angiogenesis [39]. Neutrophils and monocytes are the dominant subpopulations of infiltrating leukocytes during the early inflammatory response, producing a large number of inflammatory and pro-angiogenic cytokines, including VEGF [40]. These inflammatory chemokines amplify the cascade inducing chemotaxis of leukocytes and polarizing macrophages toward a pro-angiogenic phenotype [41]. In the present study, IMD0354 diminished CCL2 (monocyte chemoattractant protein 1), an inflammatory chemokine mediating leukocyte extravasation through the vascular endothelium [42], and CXCL5 (epithelial cell-derived neutrophil-activating peptide 78) that recruits neutrophils, promotes angiogenesis and remodels connective tissue [43, 44]. Moreover, with IKK2 blockade, we additionally observed a reduction in *Cxcr2* expression, the receptor for Cxcl5, which plays a crucial role in both angiogenesis and inflammation through neutrophil recruitment [45]. Furthermore, TNF-α, which was shown to stimulate CCL2 and CXCL5 expression by vascular endothelium in vitro, was diminished by IKK2 inhibition in the rat cornea in vivo, leading to subsequent suppression of chemokine expression and leukocyte infiltration into the corneal tissue.

Genome-wide screening in a corneal model of inflammatory neovascularization in the rat showed that *Ccl2* and *Cxcl5* were the most up-regulated factors during active angiogenesis [14]. The same factors were also shown to be significantly down-regulated by corticosteroid treatment [13]. Here, NF-κB blockade by selective IKK2 inhibition down-regulated the expression of crucial downstream chemokines, resulting in a weaker vasodilation response, reduced cellular chemotaxis and reduced infiltration of inflammatory cells into the corneal stroma. These findings in the cornea are novel, but consistent with the effects of IKK2 disruption in other tissues [20, 22, 46]. Our results in assays lacking active inflammatory components, such as HUVEC migration and tube formation, rat aortic ring sprouting and zebrafish embryonic vascular development, indicate IKK2 blockade has an inhibitory effect on endothelial cell function and VEGF-A production under physiological conditions. Reduced expression of VEGF-A as a result of NF-κB inhibition has previously been reported [47]. Our findings in zebrafish embryos retinal and intersegmental vessels development are also consistent with a recent study where in an Ikk2 mutant strain impaired angiogenesis and body axis formation was described. [48].

The mechanism of VEGF-A suppression that we hypothesize here is mediated by HIF-1α: NF-κB binds directly to an element in the proximal promoter of HIF-1α gene, increasing its expression [49]; HIF-1α is known to bind to a hypoxia-responsive element (HRE) within the promoter of VEGF gene, in this way up-regulating VEGF-A expression [50]. Moreover, one of the HIF-1α downstream targets is known to be NF-κB [51]: The positive feedback amplifies the inflammatory and angiogenic response. NF-κB inhibition decreases HIF-1α expression and consequently decreases VEGF-A [52]. A recent study reported that HIF-1α knockout mice showed a decreased VEGF production compared to wild type animals in a model of laser choroidal neovascularization (CNV) [53]. HIF-1α association with NF-κB pathway activation and IKK2 has been reported in primary endothelial cell culture settings [54]. There is also evidence that the inflammatory component independent of hypoxia may be important for HIF-1α activation [55]. Since the cornea under normal conditions is an avascular tissue, inflammation-induced hypoxia is more prominent in corneal tissue, as supported by findings in a mouse model of chronic contact lens wear [56].

**Fig. 10:**
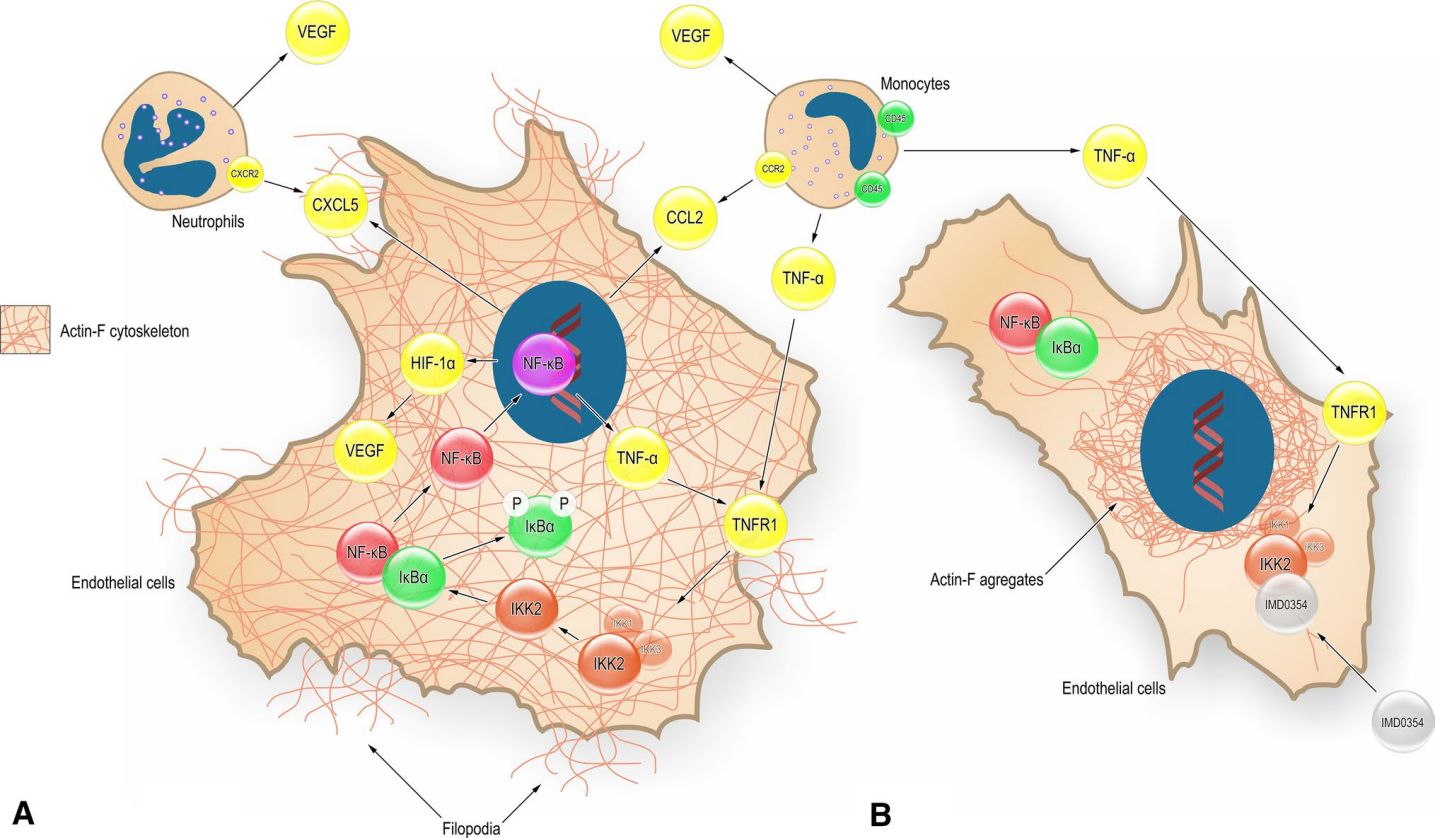
Conceptual summary of proposed cellular pathways affected by specific NF-κB blockade in the context of inflammation and angiogenesis, based on the current findings. **a** Endothelial cell (EC) is exposed to inflammatory stimulus TNF-α, which binds to TNFR1 and triggers a signal transduction resulting in phosphorylation of IκBα by IKK2; IκBα is ubiquitinated and degraded; this process enables the nuclear translocation of NF-κB. Upon nuclear translocation, NF-κB up-regulates a variety of pro-inflammatory and pro-angiogenic genes, including TNF-α, CXCL5, CCL2, and HIF-1α. TNF-α has auto- and paracrine effects and activates NF-kB through positive feedback, which amplifies the inflammatory response. CCL2 and CXCL5 have chemotactic effects on monocytes and neutrophils that in turn secrete a variety of cytokines, including TNF-α and VEGF. Nuclear NF-κB also up-regulates HIF-1α and by this mechanism can directly increase VEGF production. VEGF, in turn, acts on the EC, affecting actin polymerization, cytoskeleton composition, cell motility, tube formation and sprouting angiogenesis. **b** Selective IKK2 inhibition by IMD-0354 inhibits IκBα phosphorylation by IKK2 disrupting NF-κB activation and nuclear translocation. The pro-inflammatory and pro-angiogenic cytokine and chemokine production are substantially diminished. With decreased levels of VEGF, actin cytoskeleton formation, EC motility and migration are all suppressed. The inhibition of NF-kB also reduces the inflammatory response through suppression of the transcription of pro-inflammatory genes

The importance of IKK2 on endothelial cell function has been shown in endothelial-specific IKKβ-deleted mice where reduced migration of IKK2^−/−^ endothelial cells and involvement of IKK2 and AKT pathways were reported [57]. Our results using HUVEC indicated disruption of the microfilament meshwork and cell filopodia formation upon IMD0354 treatment. Reduced VEGF production interferes with the Actin-F meshwork and reduces endothelial cell migration; these phenomena have been reported to be mediated by a reduced activity of the stress-activated protein kinase-2/p38 (SAPK2/p38), which is VEGF induced [47]. The HIF-1α reduction may also be a contributing factor, as it has been shown that HIF-1α knockout cells had impaired migration abilities independent of VEGF or hypoxia signaling [58]. Here, we show that IMD0354 may impair migration by having a direct effect on the cell’s actin microfilament meshwork. Based on findings in the present study, the proposed multiple cellular pathways involved in the mechanism of action of IMD0354 are depicted conceptually in Fig. 10.

Despite a significant reduction in the corneal neovascularization response and a significant inhibition of multiple pro-inflammatory and pro-angiogenic factors, the selective inhibition of IKK2 could reduce the severity, but not completely prevent neovascular growth in our model. Our recently published microarray data suggest the involvement of multiple pathways in corneal neovascularization [14, 59] underscoring the complexity of this pathological condition.

As NF-κB plays a crucial role in many cellular processes including cell survival and proliferation [60], its inhibition could lead to undesirable side effects. The potential toxicity of IMD0354 was evaluated in vitro in HUVEC, as well as in vivo in zebrafish and by histological examination of the IMD0354-treated rat liver. The liver was chosen not only as a typical target for pharmacologic toxicity, but also due to its specific susceptibility to NF-κB inhibition [61]. No cell death was noted in vitro at up to 10 ng/ml concentration of IMD0354, teratogenic effects in zebrafish embryos occurred only at 10–20 times the nominal dose, while in the rat liver no histological abnormalities or increased cell death (cleaved caspase 3 signal) was detectable. This observation is in accordance with a previous study that reported no noticeable adverse effects in mice treated with IMD0354 (30 mg/kg) over 12 weeks [22]. Considering the effects of IMD0354 on the actin microfilament meshwork and its capacity to arrest and disrupt the cell cycle [62], a potential carcinogenic effect might be expected during chronic use at higher doses. However, no such effects have been reported thus far. On the contrary, several studies indicate IMD0354 has the capacity to induce cell death and reduce proliferation in cancer cells of different progeny [63–65].

At the present day, out of 780 different substances identified to have inhibitory effect on NF-κB activation, presently no FDA approved IKK2 inhibitor exists for clinical use [66]. With NF-κB being involved in multiple cellular pathways, the risk of complications frequently outweighs the benefits of disrupting the NF-κB pathway for therapeutic effect. While injection of IMD0354 may be challenging for practical clinical use, IMD1041, an oral prodrug form where IMD-0354 is the sole metabolite, appears to be more promising [65]. For IMD-1041, a Phase IIa (NCT00883584) clinical trial has been conducted. Alternatively, a formulation of IKK2 inhibitors safe for topical use is desirable for corneal neovascularization treatment, where potent local inhibition of IKK2 may be achieved with reduced risk of systemic side effects due to low systemic absorption [67]. Further studies, however, are required to identify the most suitable combination of IKK2 inhibitor, concentration and solvent medium for topical use.

## Conclusion

Our data indicate that selective inhibition of the NF-κB pathway by targeting IKK2 induces broad anti-inflammatory and anti-angiogenic activity including the suppression of NF-κB downstream inflammatory mediators and an inhibitory effect on VEGF-A, thereby impairing blood vessel formation in vitro and in vivo. In the case of corneal neovascularization, IKK2 inhibition suppressed inflammatory cell infiltration into the corneal stroma, limbal vasodilation, and the levels of crucial mediators of inflammatory angiogenesis such as VEGF-A, CCL2, CXCL5, TNF-α and expression of *Cxcr2*; in separate experiments IKK2 inhibition disrupted the microfilament meshwork and cell filopodia and reduced migration of endothelial cells. The combined effect resulted in diminished inflammation and a reduced density of neovessels in the rat cornea. NF-κB blockade through selective IKK2 inhibition, therefore, has both anti-inflammatory and anti-angiogenic activity through the disruption of multiple cellular pathways and processes, and with optimization of drug delivery, could be a potential treatment strategy for inflammatory corneal neovascularization.

## Materials and methods

### Cell culture

Human umbilical vein endothelial cells (HUVEC; Lonza, Switzerland, Basel) were used in passage 3–6. Cells were maintained in culture media (endothelial growth medium-2; consisting of endothelial basal medium (EBM)-2, supplemented with 2% fetal calf serum (FCS), hydrocortisone, FGF-2, VEGF, R3-IGF-1, ascorbic acid, EGF, GA-1000 and heparin (EGM-2 SingleQuots, Lonza, MD, US)), with additional FCS (Sigma, MO, US; to a final concentration of 5%). HUVEC were acclimatized for 1–2 days either in working media (EBM-2 with 10% FCS, 1% Pen/Strep) or starving media (EBM-2 with 1% Pen/Strep) before the experimental start. IMD0354 (Adooq Bioscience, MO, US) was dissolved in dimethylsulfoxide (DMSO; Sigma, MO, US) before usage. Cell culture images were taken with LSM700 inverted laser confocal microscope (Carl Zeiss, Oberkochen, Germany). Images were randomized, analyzed and quantified in a masked manner.

### HUVEC migration assay

To investigate the effects of IMD0354 on HUVEC migration, a razor scratch assay was conducted as previously described [25]; HUVEC were grown to confluence in a 12-well-plate in culture media followed by 24 h starvation (starvation medium). A scratch on the diameter of the well was introduced using a sterile razor. The start point was determined based on the initial imprint of the blade at the bottom of each well. Three concentrations (0, 5 and 10 ng/ml) of IMD0354 were tested, and DMSO (Sigma, MO, US) served as negative control. HUVEC were incubated for 24 h with the corresponding compounds. Images were taken, and distance of migration was measured.

### Tube formation assay

5 × 10^5^ HUVEC were seeded onto a Matrigel (Geltrex; Thermo Fisher Scientific, MA, US) pre-coated 24-well-plate and incubated in (EBM-2 with 10% FCS, 1% Pen/Strep) with IMD0354 (10 ng/ml) or DMSO (control; Sigma, MO, US). Images were taken after 24 h incubation and analyzed. Quantification of total tubule length, number of junctions and tubules was done using AngioSys 2.0 Image Analysis Software (Cellworks, Buckingham, UK).

### Rat aortic ring assay

The rat aortic ring sprouting assay was performed as previously described [68]. Briefly, tissue, extraneous fat and blood were removed from isolated aortas and cut into rings (~ 0.5 mm in length). After overnight serum starvation, aortic rings were embedded in 80 µl Matrigel (Geltrex; Thermo Fisher Scientific, MA, US) and subjected to the treatment (10 ng/ml IMD0354 or DMSO) in culture media. Culture media and the corresponding treatments were changed on the third day, and images were taken with LM Leica DMi8 (Leica Camera, Wetzlar, Germany) on the fourth day.

### Scanning electron microscopy

HUVEC surface and filopodia morphology were visualized using scanning electron microscopy (SEM). HUVEC were seeded at a density of 10^3^ cell per well into Millicell EZ slide (Millipore, Billerica, MA, US) and cultured in EBM-2 culture medium containing IMD0354 (10, 5 and 2.5 ng/ml) or DMSO (1 µl/ml) serving as negative control for 24 h. Afterward, cells were fixed for 30 min using 2% glutaraldehyde. Fixed cells were rinsed with PBS and then dehydrated by incubating in graded solutions of ethanol in water (30, 50, 75, 100%; 15 min/solution), following graded solutions of xylene in ethanol (30, 50, 70, 100%; 15 min/solution). Each sample was then allowed to air-dry overnight prior to platinum-coating (14 nm) using Agar high-resolution sputter coater (Agar Scientific, Essex, United Kingdom) and imaged using JCM-6000 NeoScope (Nikon) at high vacuum, 15 kV, 750× magnification.

### CCL2 and CXCL5 expression profile in HUVEC following recombinant human TNF-α stimulation

5 × 10^5^ HUVEC were seeded into 6-well plates, and 5 × 10^3^ HUVEC were seeded into Millicell EZ slide (Millipore) in EGM-2 and grown to 80% confluency. Cells were treated with IMD0354 10 ng/ml, following stimulation with 20 ng/ml of recombinant human TNF-α (rhTNF-α, Gibco PHC3016, Thermo Fisher Scientific). DMSO-treated and rhTNFα-stimulated HUVEC were used as positive and negative controls, respectively. Following 4 h of incubation, cells were harvested, and total RNA was extracted and purified (PureLink RNA Mini Kit; Thermo Fisher Scientific, MA, US). Total RNA amount was quantified (NanoDrop One, Thermo Fisher Scientific) and reverse transcripted to cDNA according to the manufacturer’s protocol (SuperScript VILO cDNA Synthesis Kit; Invitrogen, CA, US in SimpliAmp Thermal Cycler; Life Technology, MA, US). Gene expression analysis was performed using custom TaqMan assays for *CCL2*, *CXCL5* and *GAPDH* (Applied Biosystems). Relative expression values of target genes were normalized to *GAPDH,* and fold change was calculated using the relative quantification (2^−ΔΔCT^) method. Four biological replicates per treatment group were run with three technical replicates for each sample.

HUVEC seeded into Millicell EZ slides were fixed after 4 h in 4% paraformaldehyde (Histolab, Gothenburg, Sweden) for 10 min, permeabilized by incubating with acetone for 10 min at – 20 °C, then blocked with 2% BSA for 1 h at room temperature (RT). Samples were incubated with CCL2 (1:100; ORB36895; Biorbyt, Cambridge, UK) and CXCL5 antibody (1:100; ORB13450; Biorbyt, Cambridge, UK), then visualized (DyLight 488, 1:1000; Thermo Fisher Scientific, MA, US) and counterstained by DAPI 1:1000 (Sigma). Slides were mounted with (ProLong Diamond antifade regent; Invitrogen, Thermo Fisher Scientific, MA, US) and imaged.

### Zebrafish husbandry

Transgenic Tg(fli1a:EGFP)y^1^ zebrafish (Zebrafish International Resource Center, ZIRC; Eugene, OR, US) [69] were produced by natural mating at Linköping University (Linköping, Sweden) and maintained according to standard protocols at the Zebrafish facility as previously described [70]. Fertilized zebrafish embryos were incubated in E3 medium at 28.5 °C and analyzed according to established guidelines [71, 72]. Zebrafish experiments were conducted under ethical permit No. 89/15 at Linköping University.

### Investigation of retinal, intersegmental vessels development and Vegf-a expression in zebrafish embryos

Tg(*fli1a*:EGFP)y^1^ zebrafish embryos at 0 h post-fertilization (hpf) were seeded in the E3 buffer and treated with IMD0354 (0, 5, and 10 ng/ml in the presence of 0.1% DMSO). The embryos were euthanized with tricaine (0.5% in the E3 buffer) at 24–72 hpf. Zebrafish embryos intended for intersegmental vessel and retinal observations were mildly fixed with 1% paraformaldehyde (Histolab, Gothenburg, Sweden) for 10 min and then immediately observed. To compensate the shallow depth of field and irregularity of embryo, body *z*-stack images were acquired and rendered into a sharp composite projection image. The number of developing retinal vessels and distances from the aortic vessel to the end of developing dorsal vessels was quantified using ImageJ and averaged. Zebrafish embryos intended for immunohistochemistry were fixed for 1 h at RT in 4% formaldehyde (Histolab, Gothenburg, Sweden). Permeabilized with acetone for 30 min, blocked overnight (5% BSA), stained against Vegf-a (1:100; GTX21316; GeneTex, PA, US), visualized using a secondary antibody (DyLight 565; 1:1000; Thermo Fisher Scientific, MA, US) and mounted (ProLong Gold antifade reagent; Invitrogen, Thermo Fisher Scientific, MA, US). Images were taken with an LSM700 upright laser confocal microscope (Carl Zeiss, Oberkochen, Germany). Embryos intended for Western blot analysis were treated with 0.5% tricaine in the E3 buffer and flash frozen at − 80 °C after complete cessation of motion. Pools of 5 embryos per sample were used for lysis.

### Investigation of IMD0354 toxicity in zebrafish embryos

Forty zebrafish embryos at 0 hpf were seeded in the E3 buffer and treated with IMD0354 (0, 1, 10, 50, 100 and 200 ng/ml in the presence of 0.1% DMSO). The embryos were studied in vivo with a Nikon SMZ 1500 microscope (Nikon, Tokyo, Japan) at 24 and 72 hpf for developmental abnormalities and embryonic death. Another set of 40 normal embryos at 72 hpf was treated with the same concentrations of IMD0354 to evaluate the effects of Ikk2 inhibition after the migration of mesenchymal layers and initial development of critical organs and tissues. All surviving embryos were euthanized at 5 day post-fertilization (dpf) with tricaine (0.5% in the E3 buffer).

### In vivo corneal neovascularization model: care of animals

Twelve to sixteen week old male Wistar rats (Scanbur AB, Sollentuna, Sweden) were used. The use of animals was in accordance with the Association for Research in Vision and Ophthalmology (ARVO) Statement for the Use of Animals in Ophthalmic and Vision Research, and all procedures were approved by the Regional Animal Ethics Review Board in Linköping, Sweden (ethical permit no. 585). Animals were maintained in a licensed care facility in standard conditions (Center for Biomedical Research, University of Linköping, Sweden).

### Suture-induced inflammatory corneal neovascularization

Rats were anesthetized intraperitoneally (i.p.) with Ketanest (ketamine; 25 mg/ml, 0.4 ml, Pfizer, NY, US) and Dexdomitor (dexmedetomidine hydrochloride; 0.5 mg/ml, 0.2 ml, Orion Pharma, Hamburg, Germany). Prior to each surgical and ophthalmic imaging procedure, topical anesthesia (1% tetracaine hydrochloride, Chauvin Pharmaceuticals, Surrey, UK) was applied. Two intrastromal sutures (10-0 nylon; MANI Inc., Togichi, Japan) were placed at a distance of 1.5 mm from the temporal limbus as previously described [35, 59]. Time of suture placement was considered as *t* = 0 h. After surgery, antibiotic eye ointment was applied (Fucithalmic, fucidic acid 1%, Abcur, Sweden), anesthesia was reversed by atipamezole hydrochloride (5 mg/ml, 0.1 ml, subcutaneously; Antisedan, Orion Pharma, Hamburg, Germany) and the animals were monitored until full recovery. Animals were treated systemically with IMD0354 (200 µl, i.p., 30 mg/kg) or vehicle (0.5% sodium carboxymethylcellulose solution (CMC) diluted in PBS; Sigma, MO, US; 200 µl, i.p.) immediately before suture placement and after 48 h. The naive group (negative control) did not undergo any surgical operation or treatment. Euthanasia was performed under general anesthesia (1 ml, 60 mg/ml, intracardial injection, sodium pentobarbital (APL, Gothenburg, Sweden)) at the experimental endpoint (96 h). The vascularized area of the cornea was harvested for further analysis. Non-sutured corneal samples of similar size served as negative controls.

### Corneal neovascularization phenotype analysis

Morphological data were collected using a clinical slit-lamp camera (Micron2, Phoenix Research Laboratories, Pleasanton, USA) and in vivo confocal microscopy (IVCM; Heidelberg Retinal Tomograph III, Heidelberg Engineering, Heidelberg, Germany). Pupil dilation was achieved by tropicamide (0.5%, 5 mg/ml) before imaging. Infiltrating inflammatory cells and limbal vessel dilation were assessed with IVCM after 5, 48, and 96 h. Shortly before the experimental endpoint (96 h), slit-lamp images were taken. The degree of corneal neovascularization was scored on a numerical scale of 0–3 (Supplementary Fig. 2). Scoring was carried out independently by two masked researchers, and the images were presented in a randomized manner; the scores obtained were then averaged.

### Immunohistochemistry

HUVEC were seeded at a density of 10^3^ cell per well into Millicell EZ slide (Millipore, Billerica, MA, US). After overnight cell attachment, HUVEC were treated for 24 h with IMD0354 (10, 5, and 2.5 ng/ml or DMSO). Samples were fixed in 4% paraformaldehyde (Histolab, Gothenburg, Sweden) for 10 min, permeabilized by incubating with acetone for 10 min at − 20 °C, then blocked with 5% BSA for 1 h at RT. Samples were incubated with VEGF-a (GTX21316, 1:100; GeneTex, Simpson, PA, US) antibody, then visualized (DyLight 519, 1:500; Thermo Fisher Scientific, MA, US), mounted (ProLong Gold antifade regent; Invitrogen, Thermo Fisher Scientific, MA, US) and imaged. Actin-F cytoskeleton of HUVEC was visualized by phalloidin red staining (1:200; Thermo Fischer Scientific, MA, US).

Harvested corneal and liver tissue was fixed in 4% paraformaldehyde (Histolab), processed for paraffin embedding and sectioned (5-µm thick sections). Resulting sections were stained with hematoxylin and eosin (H&E). Sections intended for immunohistochemical analysis were rehydrated, followed by heat-induced antigen retrieval in citrate buffer (pH 6.0), and blocking. This was followed by VEGF-A (1:100; GTX21316; GeneTex, PA, US); CCL2 (1:100; ORB36895; Biorbyt, Cambridge, UK); TNF-α (1:100; BS-2081R; Bioss, MA, USA); CXCL5 (1:100; ORB13450; Biorbyt, Cambridge, UK), CD45 (ab10558; 1:150; Abcam, US); NF-κB p65 (C-20) (1:100; SC-372 Santa Cruz Biotechnology, CA, US); *p*-IκB-α (B-9) (sc-8404; 1:200; Santa Cruz Biotechnology, CA, US) or HIF-1α (NB100-479; 1:500; Novus Biologicals, UK) antibody staining. For liver toxicity evaluation, liver samples were stained for caspase-3 cleavage (D175; 1:100; Cell Signaling, MA, US). After primary incubation, stainings were visualized (DyLight 488, 1:100, Thermo Fischer Scientific, MA, US), mounted (ProLong Gold antifade reagent with DAPI (Invitrogen, Thermo Fisher Scientific, MA, US)) and imaged.

### Western blot analysis

HUVEC, Tg(*fli1a*:EGFP)y^1^ zebrafish embryos and harvested rat corneal tissue were homogenized separately using Qiagen TissueLyser LT (Qiagen, Hilden, Germany). Protein from lysates was extracted using ReadyPrep Protein Extraction Kit (supplemented with Protease and Phosphates Inhibitor Protease Halt, Bio-Rad, CA, US), and protein concentration was quantified (Qubit 3.0 Fluorometer, Thermo Fisher Scientific, MA, US). Protein extracts, separated by SDS-PAGE [Mini Protean Precast Acrylamide Gels (Bio-Rad, CA, US)], were transferred onto a PVDF membrane (Trans-Blot Turbo transfer pack, Bio-Rad) and probed with antibodies against VEGF-A (1:1000; GTX21316); CD45 (ab10558; 1:1000); *p*-IκBα (B-9) (sc-8404; 1:200) HIF-1α (NB100-479; 1:250); anti-GFP (A-11122; 1:2000; Thermo Fisher Scientific) or β-actin (PA1-21167; 1:2000; Thermo Fisher Scientific). Target protein bands were detected with HRP-conjugated IgG antibody (AP307P, 2700944, AP308P, 2688593; 1:1000; Merck Millipore, MA, USA) and visualized (Chemiluminescence Clarity Western ECL substrate; Biorad) and imaged [LAS-500 Imaging System (General Electric, CT, US)].

### RNA isolation and quantitative real-time PCR (qRT-PCR) in rat corneas

Single corneas (non-pooled) were used for qRT-PCR analysis. RNA isolation and qRT-PCR analysis were performed as described elsewhere [59]. Briefly, total RNA from homogenized (TissueLyser LT; Qiagen, Hilden, Germany) cornea tissue was extracted and purified (PureLink RNA Mini Kit; Thermo Fisher Scientific, MA, US). Total RNA amount quantified (NanoDrop One, Thermo Fisher Scientific) and reverse transcripted to cDNA according to the manufacturer’s protocol (SuperScript VILO cDNA Synthesis Kit; Invitrogen, CA, US in SimpliAmp Thermal Cycler; Life Technology, MA, US). qRT-PCR was performed using Power SYBR Green Master Mix (Thermo Fisher Scientific, MA, US) with the following rat-specific primers: *Vegf*-*a* (forward: TTGTTCAGAGCGGAGAAAGC, reverse: TTTAACTCAAGCTGCCTCGC), *Ccl2* (forward: ATGCAGTTAATGCCCCACTC, reverse: TTCCTTATTGGGGTCAGCAC), *Cxcl5* (forward: CTCAAGCTGCTCCTTTCTCG, reverse: GCGATCATTTTGGGGTTAAT), *Cxcr2* (forwards: CCAAGCTGATCAAGGAGACC, reverse: GGGGTTAAGACAGCTGTGGA) and *Gapdh* (forward: ATGGTGAAGGTCGGTGTGAA, reverse: TGACTGTGCCGTTGAACTTG) in a StepOnePlus system (Applied Biosystems, CA, US). Relative expression values of target genes were normalized to *Gapdh,* and fold change was calculated using the relative quantification (2^−ΔΔCT^) method. Four biological replicates per treatment group were run in three technical replicates.

### Statistical analysis

All values were expressed as the mean ± standard deviation (SD) for the respective groups. Statistical analyses were performed with GraphPad Prism software (https://www.graphpad.com/scientific-software/prism/). The Student’s *t* test, one-way ANOVA test with Tukey multiple comparisons, or two-way ANOVA with Bonferroni post hoc test were used. *p* value < 0.05 was considered significant. The following designations for the *p* value were used in the manuscript figures: n.s. *p* > 0.05; *p < 0.05; **p < 0.01; ***p < 0.001.

## Electronic supplementary material

Below is the link to the electronic supplementary material.

Supplementary material 1 (DOCX 1242 kb)
