# Supplementary material for: Selective IKK2 inhibitor IMD0354 disrupts NF-κB signaling to suppress corneal inflammation and angiogenesis
Source: Angiogenesis. 2018 Jan 13;21(2):267–85. doi: 10.1007/s10456-018-9594-9 (PMC5878206; doi:10.1007/s10456-018-9594-9)
Supplement: Supplementary file 1 — Supplementary material 1 (DOCX 1242 kb) [file 10456_2018_9594_MOESM1_ESM.docx]

**Supplementary Materials**

**Selective IKK2 inhibitor IMD0354 disrupts NF-κB signalling to suppress corneal inflammation and angiogenesis**

Anton Lennikov^1,2 #^, Pierfrancesco Mirabelli ^1 #^, Anthony Mukwaya^1^, Mira Schaupper^1^, Muthukumar Thangavelu^1^, Mieszko Lachota^4^, Zaheer Ali^3^, Lasse Jensen^3^, Neil Lagali^1^*

^1^Department of Ophthalmology, Institute for Clinical and Experimental Medicine, Faculty of Health Sciences, Linkoping University, Linköping, Sweden

^2^Laboratory of Biomedical Cell Technologies, School of Biomedicine, Far Eastern Federal University, Vladivostok, Russia

^3^Department of Medical and Health Sciences, Division of Cardiovascular Medicine, Linköping University, Linköping, Sweden

^4^Department of Immunology, Medical University of Warsaw, Warsaw, Poland

# Anton Lennikov and Pierfrancesco Mirabelli contributed equally to this work.

*Corresponding author:

Neil Lagali, PhD

Department of Ophthalmology

Institute for Clinical and Experimental Medicine

Faculty of Health Sciences

Linkoping University,

58183 Linköping, Sweden

Tel +46 101034658

Fax +46 101033065

[neil.lagali@liu.se](mailto:neil.lagali@liu.se)

**Supplementary Figure 1:** Semi-quantitative corneal neovascularization score.


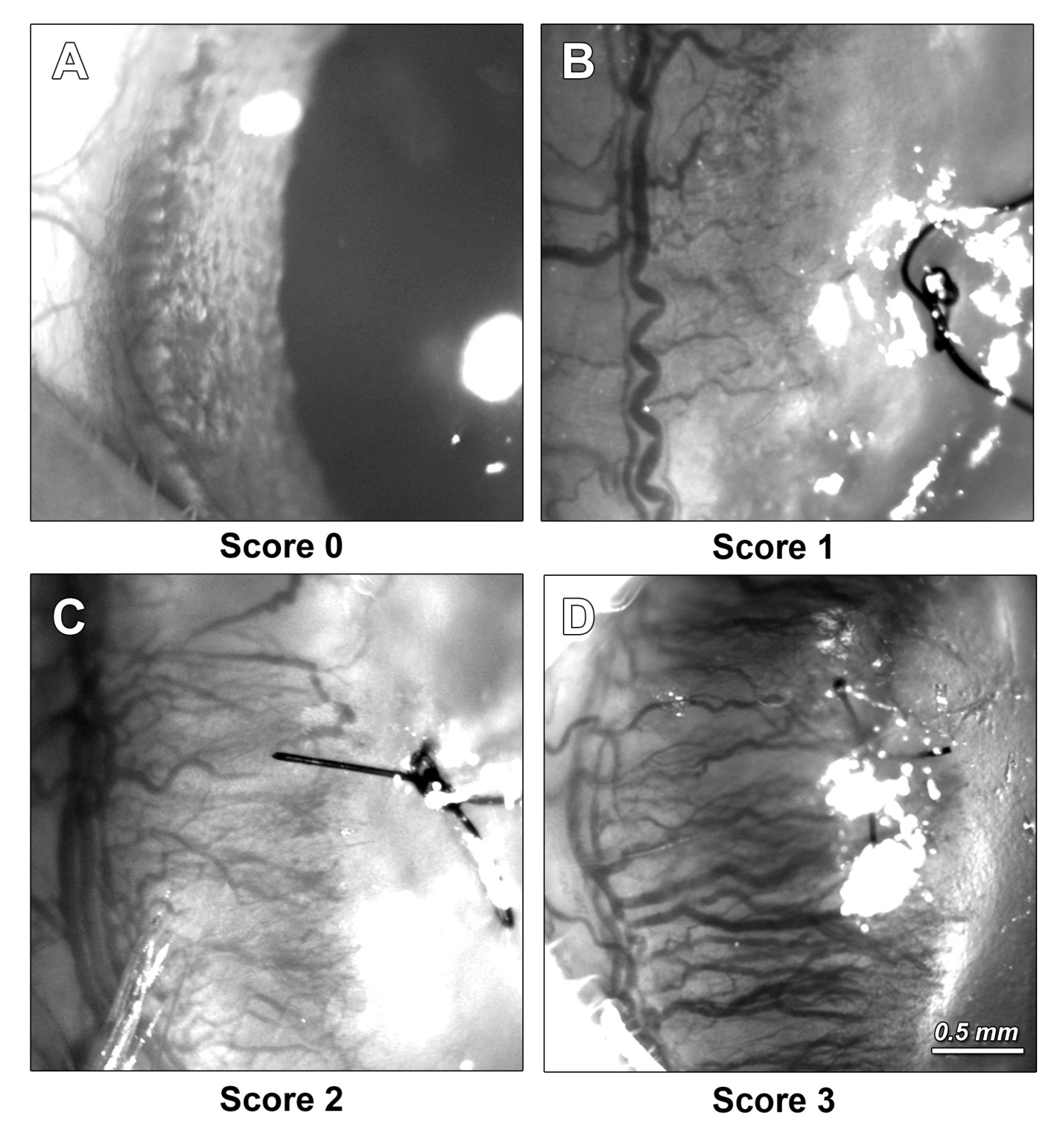


(**A**) Score 0: No new vessels sprout, minor or no dilation response from limbal vessels

(**B**) Score 1: Slight limbal vessels dilation, new vessels are visible, but do not progress more than half of the distance to suture, vessel density is low.

(**C**) Score 2: Marked limbal vessels dilation, new vessels are visible and their density is more prominent than Grade 1, vessel progress more than half of the distance to suture, but do not reach the suture.

(**D**) Score 3: Highest vessel density, and limbal vessel dilation, new vessels have reached or surpassed the suture site.

**Supplementary Figure 2:** Effect of IMD0354 treatment in zebrafish embryos and systemic effects in adult rat liver.


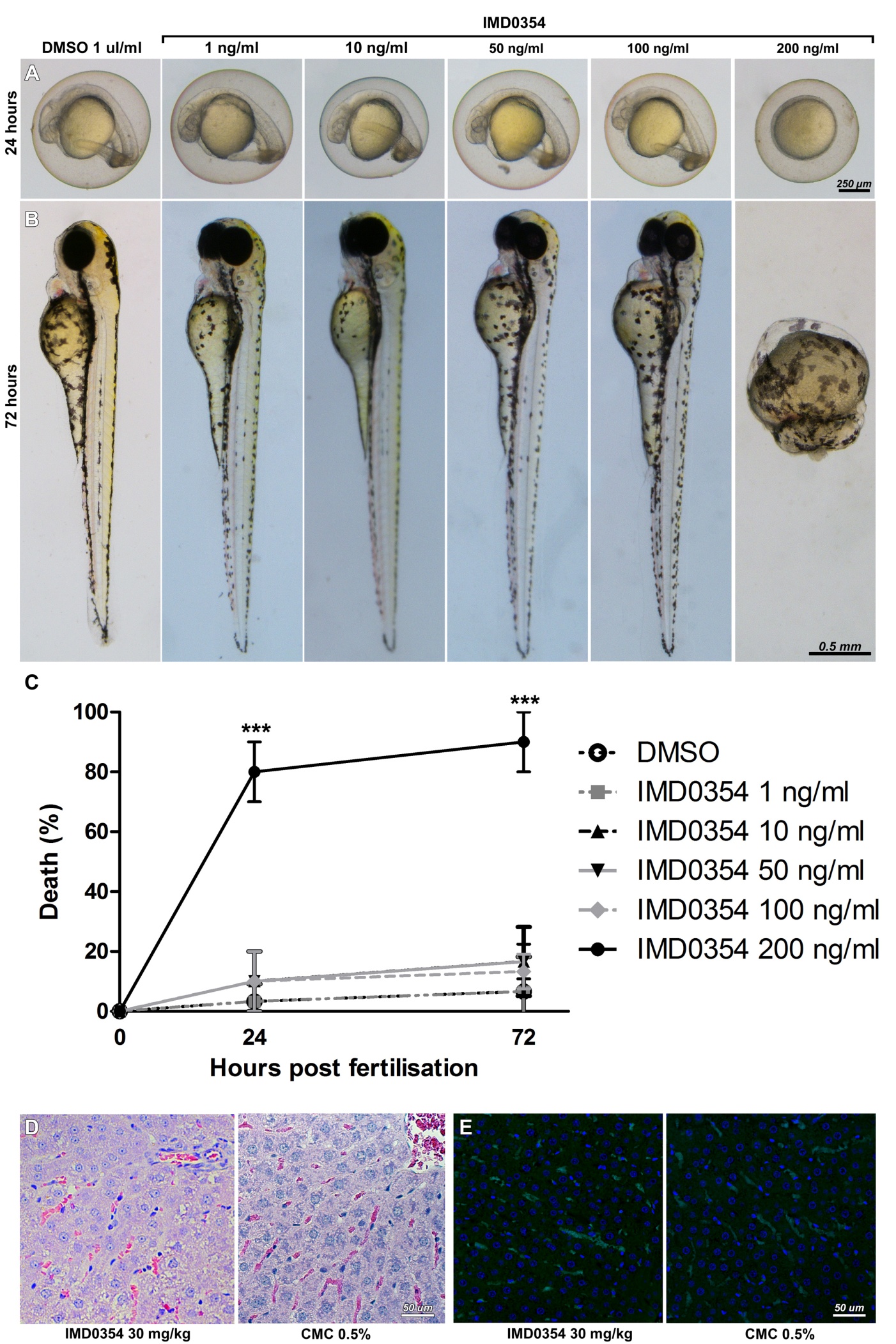


(**A**) *In vivo* images of zebrafish embryos 24 hpf, treated with IMD0354 (0, 1, 10, 100 and 200 ng/ml).

(**B**) *In vivo* images of zebrafish embryos 72 hpf, treated with IMD0354 (0, 1, 10, 100 and 200 ng/ml).

**(C)** Mortality rate of zebrafish embryos treated with IMD0354 (0, 1, 10, 100 and 200 ng/ml) at 24 and 72 hpf. (n=40) One-way ANOVA test with Tukey multiple comparisons were used to determine statistical significance. n.s. p>0.05; *** p<0.001
(**D**) Hematoxylin and Eosin (H&E), (**E**) Cleaved-Caspase 3 (green); Nuclear counterstaining by DAPI (blue) in fluorescent images.
